# Prioritizing Antidiabetic Drugs for Inflammatory Bowel Disease Through Inverse Signal Detection: A FAERS Pharmacovigilance Study

**DOI:** 10.3390/jcm15124672

**Published:** 2026-06-16

**Authors:** Katarina Đogatović, Katarina Vučićević, Srđan Marković, Milena Kovačević, Milica Ćulafić, Branislava Miljković, Sandra Vezmar Kovačević

**Affiliations:** 1 Department of Pharmacokinetics and Clinical Pharmacy, Faculty of Pharmacy, University of Belgrade, 11000 Belgrade, Serbia; 2 PrimeVigilance Ltd., 11000 Belgrade, Serbia; 3 Department of Gastroenterology and Hepatology, University Hospital Medical Center “Zvezdara”, 11000 Belgrade, Serbia; 4 Faculty of Medicine, University of Belgrade, 11000 Belgrade, Serbia

**Keywords:** drug repurposing, pharmacovigilance, signal detection, data mining

## Abstract

**Background/Objectives:** Inflammatory bowel disease (IBD) represents a growing therapeutic challenge, and the identification of novel treatment strategies remains an unmet clinical need. Drug repurposing offers a pragmatic and cost-effective alternative to de novo drug development. This study aimed to identify candidate drugs for repurposing in IBD through inverse signal detection within a large spontaneous pharmacovigilance database. **Methods:** In this observational, retrospective pharmacovigilance study, data from the FDA Adverse Event Reporting System (FAERS) were analyzed using OpenVigil 2.1. Drugs inversely associated with IBD were identified based on a ROR < 1 and an adjusted *p*-value < 0.05. Candidates were subsequently filtered to exclude agents with implausible indications, unfavorable pharmacokinetic profiles, or recognized contraindications to use in IBD. Although this screening process yielded a broader set of repurposing candidates across multiple drug classes, the present study focused specifically on antidiabetic medications, which were subjected to a targeted literature review evaluating their immunomodulatory properties, anti-inflammatory mechanisms, and existing preclinical and clinical evidence in the context of IBD. **Results:** Of 3585 initial drug–event combinations evaluated, 73 candidates met predefined criteria for statistical significance, pharmacokinetic feasibility, and clinical relevance. Within this broader pool, ten antidiabetic agents which demonstrated meaningful inverse signal strength were selected for in-depth analysis: dulaglutide (ROR 0.181, 95% CI 0.136–0.242), insulin lispro (ROR 0.206, 95% CI 0.161–0.263), insulin glargine (ROR 0.246, 95% CI 0.205–0.295), insulin (ROR 0.340, 95% CI 0.295–0.390), insulin aspart (ROR 0.349, 95% CI 0.267–0.455), empagliflozin (ROR 0.400, 95% CI 0.311–0.514), liraglutide (ROR 0.419, 95% CI 0.319–0.552), metformin (ROR 0.446, 95% CI 0.407–0.489), sitagliptin (ROR 0.460, 95% CI 0.376–0.563), and semaglutide (ROR 0.622, 95% CI 0.507–0.764). The subsequent literature review discussed relevant immunomodulatory and anti-inflammatory properties for each of these agents, providing a mechanistic rationale for their potential therapeutic role in IBD. **Conclusions:** This study identifies antidiabetic drugs as plausible repurposing candidates for IBD, supported by both pharmacovigilance-derived inverse signals and a body of mechanistic and clinical literature suggesting shared pathophysiological pathways between the two conditions. However, it should be acknowledged that the clinical evidence supporting the therapeutic efficacy of several candidates remains variable or incomplete, and robust interventional data are largely lacking. Ultimately, the findings of this study generate testable hypotheses and highlight a set of candidate therapies that warrant dedicated experimental and clinical investigation, including well-designed prospective trials, to determine their true therapeutic potential in IBD management.

## 1. Introduction

The management of inflammatory bowel disease (IBD) presents a therapeutic challenge due to its complex pathogenesis and the limitations of current immunomodulatory therapies. While biologics and small-molecule inhibitors have improved treatment, their high cost, risk of secondary non-response, and immunosuppressive side effects highlight the need for novel, safer strategies [1]. Drug repurposing offers a promising approach, particularly when guided by pharmacovigilance data capturing real-world drug–outcome associations [2]. Traditional repurposing for IBD focuses on anti-inflammatory drugs, but emerging evidence suggests that non-immunomodulatory agents, especially those targeting metabolic pathways, may offer unexpected benefits [3,4]. IBD pathophysiology—chronic inflammation, gut microbiota dysbiosis, and epithelial barrier dysfunction—overlaps with mechanisms implicated in diabetes, suggesting potential therapeutic intersections [5,6,7]. The overlap between IBD and diabetes underscores a notable comorbidity, as research indicates that the chronic inflammation associated with IBD may promote insulin resistance and elevate the risk of developing type 2 diabetes [8]. Studies show that diabetes is more common among individuals with IBD than in the general population, highlighting the importance of coordinated care to address these closely linked conditions [9].

Antidiabetic drugs have emerged as promising candidates for inflammatory bowel disease (IBD) treatment beyond their glucose-lowering effects. In particular, metformin and glucagon-like peptide-1 (GLP-1) receptor agonists have demonstrated anti-inflammatory, barrier-protective, and microbiome-modulating properties in preclinical and clinical studies, suggesting potential therapeutic benefits in IBD [5,10]. Their established safety profiles, widespread clinical use, and relevance in patients with comorbid metabolic disorders further support their attractiveness for drug repurposing.

Therefore, the objective of this study was to identify antidiabetic drugs with potential therapeutic utility in IBD using inverse signal detection within the FDA Adverse Event Reporting System (FAERS). By applying a pharmacovigilance-based data mining approach, we sought to detect inverse associations between antidiabetic drug exposure and IBD-related adverse event reporting, generating hypotheses for drug repurposing and future validation.

## 2. Materials and Methods

We conducted a retrospective pharmacovigilance analysis using the FDA Adverse Event Reporting System (FAERS) database covering the period from 2003Q4 to 2024Q3. Data mining was performed using OpenVigil 2.1. Cases were retrieved based on MedDRA version 24.0 preferred terms (PTs) corresponding to “Ulcerative colitis” and “Crohn’s disease.” As implemented in OpenVigil, querying at the PT level automatically includes all lower-level terms (LLTs) related to the selected PT in the MedDRA hierarchy; therefore, no independent LLT selection was performed. The complete list of LLTs included through the selected PTs is provided in Supplementary Table S1. A specific role of the drug in the drug–event relationship (e.g., primary suspect or concomitant) was not predefined. This approach was adopted to generate a broader background case set for each drug, reflecting the exploratory nature of inverse signal detection and acknowledging that drug role assignment in FAERS is reporter-dependent and not standardized.

Drug–event associations were evaluated using disproportionality analysis based on the Reporting Odds Ratio (ROR) with two-sided 95% confidence intervals [11]. To account for multiple comparisons, *p*-values were corrected using the Benjamini–Hochberg procedure to control the false discovery rate. Inverse pharmacovigilance signals were considered eligible when all of the following predefined criteria were simultaneously satisfied: a reporting odds ratio (ROR) below 1, a Benjamini–Hochberg-adjusted *p*-value (*p*adj) < 0.05, and a minimum of 40 reports per drug–event combination (*n* ≥ 40). A ROR below unity indicates that a given drug–event pair is reported at a lower frequency than would be expected under the null hypothesis of no association, which may reflect a protective or inverse relationship between drug exposure and the outcome of interest.

A conservative case threshold was deliberately applied to mitigate instability arising from sparse data. Although conventional disproportionality analyses typically accept minimum counts as low as three to five reports, higher thresholds are widely adopted in exploratory pharmacovigilance and drug repurposing contexts to enhance the robustness of effect estimates. Given the particular susceptibility of inverse signals to small-number bias, the present analysis prioritized estimate stability and specificity over maximal sensitivity.

Data preprocessing was performed within the OpenVigil platform and encompassed automated normalization and deduplication of raw FAERS records. Combination drug products were decomposed into their constituent active ingredients, and duplicate reports were managed in accordance with FDA-recommended case version control procedures. Reports containing drug entries that could not be unambiguously mapped to a standardized drug name were excluded from further analysis.

To enhance the clinical interpretability of detected signals, a predefined clinical feasibility filter was applied following statistical screening. Specifically, non-systemic formulations, diagnostic agents, withdrawn or discontinued products, pharmacokinetically implausible compounds, and drugs with documented lack of efficacy or known disease-aggravating properties in IBD were excluded. These exclusions were determined independently of observed signal magnitude and were applied solely to ensure the clinical plausibility of candidate compounds. Importantly, no mechanistic plausibility criteria were imposed during the statistical screening phase; mechanistic considerations were reserved for the post-hoc contextualization of identified signals.

Following statistical screening, candidate drugs were stratified according to therapeutic class. Although multiple drug classes were considered as candidates, the scope of the present analysis was limited to antidiabetic agents, due to the mechanistic links to IBD, relevance to patients with comorbid metabolic disorders, and potential to offer therapeutic benefits beyond the traditional drug classes typically used in IBD treatment (i.e., monoclonal antibodies). For the present analysis, a targeted literature review was conducted to appraise the available evidence for a selected subset of candidates, with particular emphasis on antidiabetic agents. The review focused on the antidiabetic agents identified through inverse signal detection analysis, including dulaglutide, insulin lispro, insulin glargine, insulin, insulin aspart, empagliflozin, liraglutide, metformin, sitagliptin, and semaglutide. Following identification of these candidates through inverse signal detection, literature searches were performed in PubMed to identify relevant preclinical, mechanistic, and clinical studies. Search terms consisted of the individual drug names combined with terms including “inflammatory bowel disease”, “Crohn’s disease”, “ulcerative colitis”, “colitis” and “intestinal inflammation”. Studies reporting experimental, mechanistic, observational, interventional, or case-based evidence relevant to intestinal inflammation or IBD were considered eligible for inclusion. The studies identified through this targeted literature review are summarized descriptively in Table 1. The objective of the review was to assess biological plausibility and contextualize the pharmacovigilance findings rather than to perform a formal systematic review. Preclinical evidence was defined as experimental data derived from in vitro systems or animal models relevant to intestinal inflammation or immune modulation. Clinical evidence encompassed human data from randomized controlled trials, observational studies, or case reports pertaining to IBD or related inflammatory conditions. All retrieved evidence was synthesized descriptively and was not used as a formal inclusion or exclusion criterion in the signal detection workflow.

This study was exempt from ethics committee review, as all analyses were conducted exclusively on publicly available, fully anonymized data obtained from the FDA Adverse Event Reporting System (FAERS).

## 3. Results

### 3.1. Identification of Drug Candidates

**Figure 1:**
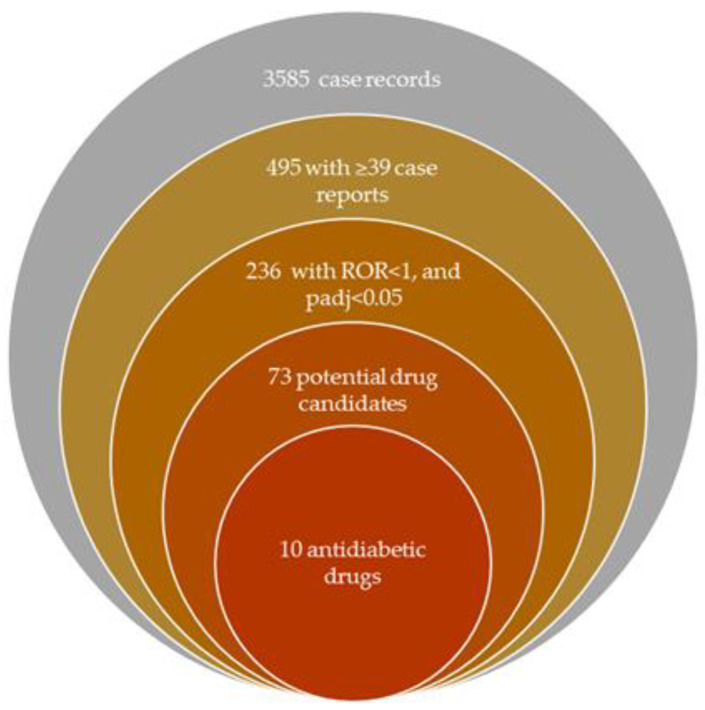
Drug candidate selection process.

A total of 3585 drug–event combinations were retrieved, out of which 495 met the minimum requirement of having more than 39 case reports. Applying additional criteria, we identified 236 drugs that fulfilled the conditions of a ROR < 1, and *p*adj < 0.05. To refine the list further, we eliminated brand names, undetected salt forms, drugs that were pharmacokinetically infeasible, drugs without systemic effects, drugs withdrawn from the market, drugs known to worsen symptoms of IBD, potentially impact disease progression, increase relapse/flares (PPIs, antibiotics, oral contraceptives), or have immunomodulatory effects that may complicate IBD management, and drugs known to be ineffective for the indication. This rigorous selection process resulted in a list of 73 potential drug candidates [32] of which 10 were antidiabetic agents as presented in Figure 1.

### 3.2. Inverse Disproportionality Analysis of Antidiabetic Agents

**Figure 2:**
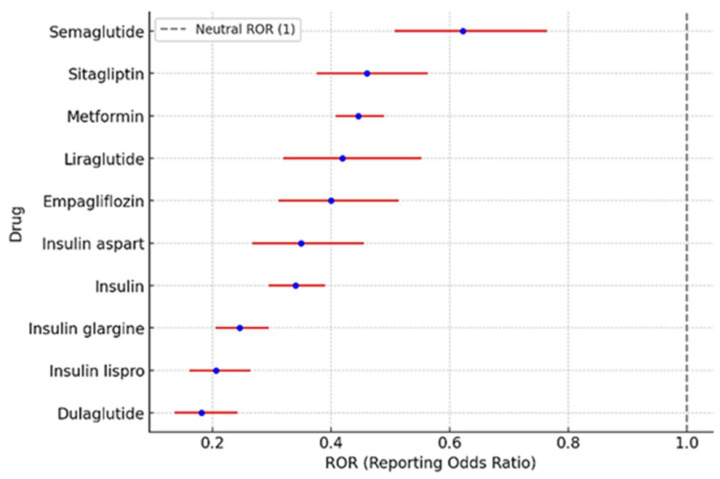
Forest plot of Reporting Odds Ratios (RORs) with 95% confidence intervals (CIs) of selected antidiabetic drugs.

Ten antidiabetic drugs which fulfilled the predefined criteria and were selected for further evaluation are shown in a forest plot diagram in Figure 2, which visualizes their confidence intervals to indicate the strength and directions of their associations.

The strongest inverse association was observed for dulaglutide (ROR 0.181, 95% CI 0.136–0.242), followed by insulin lispro (ROR 0.206, 95% CI 0.161–0.263) insulin glargine (ROR 0.246, 95% CI 0.205–0.295), insulin (ROR 0.340, 95% CI 0.295–0.390) and insulin aspart (ROR 0.349, 95% CI 0.267–0.455).

Among non-insulin agents, inverse signals were observed for empagliflozin (ROR 0.400, 95% CI 0.311–0.514), liraglutide (ROR 0.419, 95% CI 0.319–0.552), metformin (ROR 0.446, 95% CI 0.407–0.489), sitagliptin (ROR 0.460, 95% CI 0.376–0.563), and semaglutide (ROR 0.622, 95% CI 0.507–0.764).

When grouped by pharmacological class, glucagon-like peptide-1 receptor agonists (dulaglutide, liraglutide, semaglutide), insulin preparations, sodium-glucose cotransporter-2 inhibition (empagliflozin), biguanide therapy (metformin), and dipeptidyl peptidase-4 inhibition (sitagliptin) were all represented (Table 2).

Although multiple antidiabetic classes demonstrated inverse disproportionality signals, with the exception of dulaglutide which exhibited the strongest inverse association, insulin preparations exhibited the lowest inverse ROR estimates, while metformin demonstrated a comparatively narrow confidence interval, suggesting greater precision of estimation.

## 4. Discussion

This study introduces a novel, data-driven approach to identifying potential candidates for drug repurposing. In the present study, the methodology was applied to IBD with focus on antidiabetic agents, and identified several antidiabetic agents that may influence pathways relevant to diseases outside of diabetes, such as inflammation, cardiovascular dysfunction, or neurodegeneration. These findings highlight the broader therapeutic potential of antidiabetic drugs and highlight their possible roles in managing comorbidities or entirely distinct diseases. Overall, antidiabetic agents identified here demonstrate promising anti-inflammatory and immunomodulatory effects in IBD, with consistent preclinical evidence across insulin, oral antidiabetics, and Glucagon-Like Peptide-1 (GLP-1) receptor agonists, primarily through modulation of immune pathways, gut microbiota, and epithelial barrier integrity. However, clinical evidence remains limited and heterogeneous, with metformin showing the most consistent benefit, while other candidates require robust controlled trials to confirm their therapeutic potential. The preclinical and clinical evidence supporting the repurposing potential of the selected antidiabetic agents is summarized in Table 1.

Insulin and certain antidiabetic drugs, such as metformin, may exhibit anti-inflammatory properties, potentially reducing inflammation in IBD [5,12]. Emerging evidence suggests that insulin and antidiabetic drugs may influence the gut microbiota, which plays a crucial role in IBD pathogenesis [5,6,13]. Modulating the gut microbiota could potentially contribute to improved disease outcomes [5,6]. However, both IBD and diabetes share certain risk factors, such as genetic predisposition, obesity, and chronic inflammation [6,7,8,9]. These shared risk factors could potentially complicate the analysis of the association between insulin/antidiabetic drug use and IBD. The immune system dysregulation observed in both IBD and diabetes suggests a potential interaction between the two conditions [6,7]. It is essential to consider the potential confounding effects of other medications commonly used in IBD management, such as corticosteroids or immunosuppressants, which may impact the analysis of insulin/antidiabetic drug use in IBD.

### 4.1. Insulin and Insulin Analogues: Insulin Lispro, Insulin Glargine, Insulin, Insulin Aspart

Insulin may play an immunomodulatory role in anti-inflammation by regulating the NOD-, LRR-, and Pyrin Domain-Containing Protein 3 (NLRP3) inflammasome [12]. Low-dose insulin alleviated intestinal inflammation in murine IBD models through a gut microbiota-dependent mechanism, increasing lithocholic acid (LCA) levels, which activated Takeda G Protein-Coupled Receptor 5 (TGR5) to inhibit pro-inflammatory classically activated (M1) macrophage polarization [13]. An interesting therapeutic approach with potential relevance for patients with active UC is the local rectal administration of insulin, which demonstrated promising effects as monotherapy in the azoxymethane/dextran sodium sulfate (AOM/DSS) model of chronic colitis and colitis-associated carcinogenesis [14]. In this setting, rectally delivered insulin was associated with reduced mucosal inflammation, improved epithelial barrier integrity, and decreased tumor development, suggesting benefits beyond glycaemic regulation through promotion of epithelial restitution and modulation of inflammatory pathways. Given the importance of barrier dysfunction in UC pathogenesis, localized insulin delivery may represent a particularly attractive strategy by achieving high concentrations at the site of inflammation while limiting systemic exposure.

### 4.2. Oral Antidiabetic Medications: Metformin, Empagliflozin, Sitagliptin

Metformin use in patients with type 2 diabetes mellitus (T2DM) and IBD is associated with improved outcomes, such as a reduced need for intravenous steroids and a lower risk of IBD-related surgeries, particularly in CD [15]. Additionally, metformin use is linked to a lower risk of developing IBD in T2DM patients [16]. Metformin activates AMP-activated protein kinase (AMPK), which helps regulate immune cells and reduce inflammation in the gut as shown in a chronic colitis mouse model [17]. In an in vitro study on mouse colon smooth muscle cells, metformin reduced the expression of pro-inflammatory cytokines such as Tumor Necrosis Factor Alpha (TNF-α) and Interleukin-1 Alpha (IL-1α) in colon smooth muscle cells, contributing to its anti-inflammatory effects [18]. Furthermore, metformin suppresses the Signal Transducer and Activator of Transcription 3 (STAT3) signaling pathway, which is involved in inflammatory processes, thereby reducing inflammation in IBD [19].

In vitro experiments show that Empagliflozin suppresses nitric oxide production in Lipopolysaccharide (LPS)-treated murine RAW264.7 macrophages, and in vivo studies reveal it alleviates acute DSS-induced colitis in mice, reduces macroscopic and microscopic colonic damage, attenuates biochemical inflammatory parameters including reduced activity of myeloperoxidase [20]. Furthermore, successful Crohn disease-like enterocolitis remission was reported after empagliflozin treatment in a child with glycogen storage disease type Ib (GSD-Ib) [21]. Another case was reported of an adult patient with GSD-Ib whose neutropenia and IBD-related symptoms were successfully treated with empagliflozin. The mechanism by which empagliflozin improves IBD-related disease remains unclear. However, it is suggested that overall improved leukocyte function may positively influence the inflammatory pathways involved in IBD pathophysiology [22]. Regarding sitagliptin, results of one study demonstrate that it could attenuate DSS-induced experimental colitis by enhancing Glucagon-Like Peptide-2 (GLP-2) action and the subsequent protective effects on intestinal barrier by inhibiting epithelial cell apoptosis and promoting their proliferation. These findings suggest sitagliptin as a novel therapeutic approach for the treatment of ulcerative colitis [23]. A population-based cohort study suggested that the use of dipeptidyl peptidase-4 inhibitors was linked to an increased risk of inflammatory bowel disease [24]. However, a later meta-analysis invalidated these findings [25].

### 4.3. GLP-1 Receptor Agonists: Dulaglutide, Liraglutide, Semaglutide

Recent preclinical studies have demonstrated that GLP-1 receptor agonists (GLP-1RA), including dulaglutide, liraglutide, and exendin-4, exhibit potent anti-inflammatory effects in models of inflammatory bowel disease. In rats with acetic acid-induced ulcerative colitis, dulaglutide significantly reduced histological inflammation and levels of C-Reactive Protein (CRP) and lactate dehydrogenase (LDH) while inhibiting Transforming Growth Factor Beta 1 (TGF-β1)/Phosphoinositide 3-Kinase (PI3K)/Protein Kinase B (AKT)/Nuclear Factor Kappa B (NF-κB) signaling, boosting antioxidant defenses, enhancing trefoil factor-3 and GLP-1 expression, and preserving mucosal integrity [26]. Liraglutide has been shown to improve disease activity, colon length, and histological scores in DSS-induced colitis in mice, in a mechanism dependent on IL-22-producing group 3 innate lymphoid cells (ILC3s) [27]. A clinical report describes a patient with ulcerative colitis achieving full remission after 8 weeks of liraglutide treatment [28]. Another study highlights the potential of GLP-based therapy as an adjunct to sulfasalazine in improving IBD in an acetic acid (A-A) mouse model. This effect is achieved by regulating NFκB-dependent inflammatory cytokine secretion, reducing oxidative stress injury, and promoting tissue repair of the injured epithelium, with better-tolerated side effects [29]. Additionally, a small retrospective study of IBD patients treated with GLP-1RAs observed a significant decrease in CRP at one year, indicating an anti-inflammatory effect though without clear changes in clinical scores [30]. Although semaglutide is effective for weight loss in non-diabetic patients with IBD, its potential to alter the disease course remains largely unknown [31].

The identification of antidiabetic agents as a cluster of inverse signals is particularly noteworthy given the growing recognition of interactions between metabolic dysfunction, immune regulation, gut microbiota composition, and intestinal inflammation [5,6,7]. Several of the identified agents demonstrated overlapping mechanisms involving modulation of inflammatory signaling pathways, enhancement of epithelial barrier integrity, and regulation of microbiota-derived metabolites. The convergence of independent pharmacovigilance signals with emerging experimental evidence suggests that metabolic pathways may represent an underexplored therapeutic target in inflammatory bowel disease and supports further investigation of these agents in prospective studies [3,5,6,7].

Despite the inherent limitations of spontaneous reporting systems, several strengths of the present analysis warrant consideration. First, this study utilized FAERS, one of the largest pharmacovigilance databases globally, encompassing more than two decades of real-world safety data and millions of reports. Second, the application of inverse signal detection extends the traditional role of pharmacovigilance beyond adverse event identification and provides a systematic framework for generating drug repurposing hypotheses. Third, the use of conservative signal detection criteria, including adjustment for multiple testing and a minimum case threshold, improved the robustness of the identified associations and reduced the likelihood of spurious findings arising from sparse reporting.

An additional strength is the incorporation of clinical feasibility filtering following statistical signal detection. By excluding non-systemic therapies, withdrawn products, agents with known disease-aggravating effects, and pharmacokinetically implausible candidates, the analysis prioritized compounds with greater translational potential. Furthermore, the convergence of inverse pharmacovigilance signals with existing mechanistic, preclinical, and clinical evidence for several antidiabetic agents provides additional support for the biological plausibility of the findings.

The present analysis draws on data from a spontaneous adverse event reporting system, and its findings must be interpreted within the inherent methodological constraints of this data source. Spontaneous pharmacovigilance databases such as FAERS are subject to well-recognized limitations including underreporting, selective and stimulated reporting, heterogeneity in reporting practices across countries and healthcare systems, and confounding by indication. Inverse disproportionality signals identified through this approach may therefore reflect patterns of drug utilization, comorbidity distributions, or systematic reporting behavior rather than genuine protective biological effects. Critically, spontaneous reporting systems were designed to detect potential safety signals rather than to evaluate therapeutic efficacy, and any associations suggesting a protective role should accordingly be framed within the context of hypothesis generation rather than interpreted as evidence of clinical benefit.

A fundamental limitation of the FAERS database is the absence of reliable exposure denominators. Because the database captures reports of suspected adverse drug reactions without providing information on the total number of patients exposed to a given medication in the underlying population, disproportionality analyses are incapable of estimating incidence rates or absolute risk. Reporting Odds Ratios therefore represent relative reporting frequencies within the database rather than true population-level risk estimates. Differences in prescribing practices, drug utilization patterns, and reporting behavior may independently influence observed disproportionality signals, irrespective of any pharmacological effect. Inverse associations derived from this framework must consequently be interpreted with caution and require validation through epidemiological studies employing well-defined exposure data.

FAERS also lacks the comprehensive clinical covariate data necessary to adjust for important confounding factors. Information regarding patient medical history, disease severity, treatment duration, and concomitant medication use is frequently incomplete or entirely unavailable. Case narratives, time-to-onset data, and documentation of dechallenge or rechallenge are inconsistently recorded across reports. In the absence of reliable temporal information, it cannot be established whether drug exposure preceded disease onset, modified disease course, or occurred independently of the reported outcome. The observed associations should therefore be regarded as hypothesis-generating and not as indicators of therapeutic efficacy.

Several drug classes demonstrating inverse signals in this analysis including statins and antihypertensive agents are disproportionately prescribed in older patients with metabolic or cardiovascular comorbidities. Their apparent inverse disproportionality may consequently reflect demographic clustering, comorbidity-driven prescribing, or channeling bias rather than any direct biological effect on inflammatory bowel disease. Polypharmacy is additionally common in the FAERS reporting population, and co-medication patterns may substantially influence disproportionality estimates. Because adjustment for concomitant drug exposure was not feasible within this analytical framework, some inverse signals may represent complex prescribing patterns rather than independent pharmacological effects.

A further methodological consideration concerns the classification of drug–event relationships within spontaneous reports. In conventional pharmacovigilance, analyses are often restricted to drugs designated as “suspect,” as these are considered most likely to have contributed causally to the reported event. Applying this restriction to the analysis of inverse signals would, however, introduce systematic bias: drugs with a potential protective effect are unlikely to be reported as causative and would therefore be excluded under such a framework. To mitigate this, the present analysis included all reported drugs regardless of their assigned role. While this approach may increase background noise through non-causal co-reporting, restricting the analysis to suspect drugs would disproportionately exclude chronic background therapies and distort exposure patterns in immune-mediated conditions. Nonetheless, some drugs may be consistently co-prescribed with agents known to cause gastrointestinal adverse events, potentially creating artifactual reporting patterns that are independent of any true protective effect.

Beyond the limitations outlined above, several additional constraints are specific to the use of FAERS for inverse signal detection and deserve explicit consideration. First, the Weber effect, a well-documented phenomenon in which reporting rates for a given drug are highest in the period immediately following market authorization and decline thereafter, may differentially affect drugs at various stages of their lifecycle. Newly marketed agents may generate disproportionately high reporting volumes regardless of their true pharmacological profile, while older, more established drugs may be systematically underrepresented, potentially distorting inverse associations in ways that are unrelated to biological effect.

Second, notoriety bias and stimulated reporting present particular challenges in this context. High-profile safety communications, regulatory actions, or media attention surrounding specific drugs can precipitate marked and transient increases in reporting for selected drug–event combinations, inflating disproportionality estimates and potentially masking or generating inverse signals for co-reported medications.

Third, duplicate reporting represents an underappreciated source of bias in FAERS-based analyses. Although deduplication procedures were applied, FAERS contains a substantial proportion of duplicate or near-duplicate records arising from parallel submissions by manufacturers, healthcare professionals, and consumers. Residual duplicates, particularly for widely used drugs, may artificially inflate the frequency of certain drug–event pairs and distort the denominators used in disproportionality calculations, with unpredictable consequences for inverse signal detection. Fourth, the geographic and institutional heterogeneity of FAERS submissions requires consideration. Reporting rates, drug availability, prescribing norms, and disease prevalence vary substantially across contributing regions, and the database is heavily weighted toward reports originating from the United States. Inverse signals identified in this analysis may therefore not generalize to other healthcare settings or patient populations.

Fifth, in the specific context of inflammatory bowel disease, a condition characterized by relapsing and remitting disease activity and complex, often evolving treatment regimens, the limitations of cross-sectional snapshot data are particularly pronounced. FAERS does not capture longitudinal treatment histories, and a single report may reflect only a fragment of a patient’s therapeutic journey. The absence of information on disease phenotype, prior treatment exposure, and surgical history further limits the interpretability of observed signals. Finally, it should be noted that inverse disproportionality is a mathematically derived reporting artifact as much as it is a pharmacological signal: a drug may appear inversely associated with a condition simply because it is frequently reported in the context of other outcomes, thereby diluting its apparent co-occurrence with the event of interest. This statistical phenomenon, sometimes described as masking or the submersion effect, is particularly relevant when analyzing drugs with broad therapeutic indications and high overall reporting volumes.

Taken together, these limitations highlight that the findings of inverse signal detection within a spontaneous reporting database should be viewed as an exploratory, hypothesis-generating starting point for further investigation rather than as definitive evidence of therapeutic benefit.

Validation of the associations identified here requires mechanistic investigation, pharmacoepidemiological analyses with controlled designs and clearly defined exposure and outcome ascertainment, and ultimately prospective clinical evaluation to determine whether the identified candidates demonstrate meaningful efficacy and an acceptable safety profile in inflammatory bowel disease. Nevertheless, the approach described here offers a pragmatic and cost-effective complement to conventional drug development pathways. By leveraging existing real-world safety data to identify repurposing candidates, this strategy may help accelerate therapeutic discovery, particularly for conditions in which treatment options remain limited. Importantly, the concordance observed between several inverse pharmacovigilance signals and published mechanistic or clinical evidence supports the utility of inverse signal detection as a hypothesis-generating tool for drug repurposing research. Future studies should focus on validating these findings through mechanistic investigations, pharmacoepidemiological analyses, and prospective clinical evaluation.

## 5. Conclusions

This study demonstrates the potential of a systematic, data-driven approach to identify alternative candidate drugs for IBD, with a particular focus on antidiabetic medications. By leveraging real-world pharmacovigilance data, we uncovered promising inverse signals that suggest broader therapeutic roles for these drugs beyond glycemic control. To our knowledge, the application of inverse disproportionality analysis within the FAERS database for the purpose of antidiabetic drug repurposing in IBD represents a novel methodological contribution, distinguishing this work from conventional pharmacovigilance studies that focus exclusively on adverse event detection. While the inherent limitations of spontaneous reporting databases, including reporting bias, confounding, and the inability to establish causality, must be acknowledged, the present framework is explicitly hypothesis-generating in nature. As such, the (inverse) signals identified are intended to prioritize candidates for subsequent mechanistic and clinical investigation rather than to provide confirmatory evidence of therapeutic efficacy. Looking ahead, further studies exploring the underlying mechanisms and clinical effectiveness of these candidate drugs will be essential. Repurposing drugs with established safety profiles not only accelerates therapeutic discovery but also supports the development of more personalized and targeted treatments. Moreover, this approach serves as a scalable framework that can be extended to other drug classes and disease areas, offering a reproducible and resource-efficient strategy for systematic drug repurposing.

## Figures and Tables

**Table 1:** Summary of the mechanistic rationale and preclinical/clinical evidence for prioritized candidates—antidiabetic drugs.

Citation	Drug/Class	Preclinical Evidence	Clinical Evidence	Summary of Evidence
Chang et al., 2021 [12]; He et al., 2023 [13]; Yassin et al., 2018 [14]	Insulin & analogues	Anti-inflammatory effects via NLRP3 inhibition, microbiota modulation, and improved epithelial barrier in murine models	No robust clinical studies	Strong mechanistic evidence but lacks clinical validation
Petrov et al., 2024 [15]; Tseng, 2020 [16]; Takahara et al., 2022 [17]; Al Dwairi et al., 2018 [18]; Lee et al., 2015 [19]	Metformin	AMPK activation, reduced pro-inflammatory cytokines, STAT3 inhibition	Reduced steroid use, surgery risk, and IBD incidence in observational studies	Most consistent candidate with both mechanistic and clinical support
Makaro et al., 2023 [20]; Rossi et al., 2021 [21]; Makrilakis et al., 2022 [22]	Empagliflozin	Reduced NO and MPO, improved colitis in DSS models	Case reports showing remission in GSD-Ib patients	Promising but limited clinical evidence
Ning et al., 2020 [23]; Abrahami et al., 2018 [24]; Radel et al., 2019 [25]	Sitagliptin	Improved epithelial integrity via GLP-2, reduced apoptosis	Conflicting evidence on IBD risk	Uncertain net effect
El Mahdy et al., 2024 [26]; Sun et al., 2024 [27]; Jeffrey, 2019 [28]; Azmy et al., 2024 [29]; Coats et al., 2024 [30]; St-Pierre et al., 2024 [31]	GLP-1 receptor agonists	Reduced inflammation, NF-κB inhibition, improved mucosal healing	Case reports and small studies showing CRP reduction	Strong preclinical and early clinical signals; needs RCTs

Abbreviations: AMPK, adenosine monophosphate-activated protein kinase; CRP, C-reactive protein; DSS, dextran sulfate sodium; GLP-1, glucagon-like peptide-1; GLP-2, glucagon-like peptide-2; GSD-Ib, glycogen storage disease type Ib; IBD, inflammatory bowel disease; MPO, myeloperoxidase; NF-κB, nuclear factor kappa B; NLRP3, NOD-, LRR- and pyrin domain-containing protein 3; NO, nitric oxide; RCTs, randomized controlled trials; STAT3, signal transducer and activator of transcription 3.

**Table 2:** Pharmacological classification and inverse disproportionality results of antidiabetic agents identified as potential candidates for inflammatory bowel disease repurposing.

Pharmacological Class	Drug	ROR	95% CI
GLP-1 receptor agonists	Dulaglutide	0.181	0.136–0.242
	Liraglutide	0.419	0.319–0.552
	Semaglutide	0.622	0.507–0.764
Insulin preparations	Insulin lispro	0.206	0.161–0.263
	Insulin glargine	0.246	0.205–0.295
	Insulin	0.340	0.295–0.390
	Insulin aspart	0.349	0.267–0.455
SGLT2 inhibitors	Empagliflozin	0.400	0.311–0.514
Biguanides	Metformin	0.446	0.407–0.489
DPP-4 inhibitors	Sitagliptin	0.460	0.376–0.563

Abbreviations: ROR, reporting odds ratio; CI, confidence interval; GLP-1, glucagon-like peptide-1; SGLT2, sodium-glucose cotransporter-2; DPP-4, dipeptidyl peptidase-4.

## Data Availability

The FDA Adverse Event Reporting System (FAERS) database and source are freely available. OpenFDA is freely accessible at https://api.fda.gov/drug/event.json (accessed on 24 December 2025). OpenVigil FDA can be used or downloaded at http://openvigil.sourceforge.net (accessed on 24 December 2025).

## Supplementary Materials

The following supporting information can be downloaded at: https://www.mdpi.com/article/10.3390/jcm15124672/s1, Table S1: MedDRA v24.0 preferred terms (PTs) and associated lower-level terms (LLTs) used for case retrieval.

## Abbreviations

The following abbreviations are used in this manuscript:

AOM	Azoxymethane
AMPK	AMP-activated protein kinase
CD	Crohn’s disease
CRP	C-reactive protein
DPP-4	Dipeptidyl peptidase-4
DSS	Dextran sulfate sodium
FAERS	FDA Adverse Event Reporting System
GLP-1	Glucagon-like peptide-1
GLP-1RA	Glucagon-like peptide-1 receptor agonist
GLP-2	Glucagon-like peptide-2
GSD-Ib	Glycogen storage disease type Ib
IBD	Inflammatory bowel disease
IL	Interleukin
ILC3	Group 3 innate lymphoid cells
LCA	Lithocholic acid
LDH	Lactate dehydrogenase
LLT	Lower-level term
LPS	Lipopolysaccharide
MedDRA	Inflammatory bowel disease
MPO	Myeloperoxidase
NF-κB	Nuclear factor kappa B
NLRP3	NOD-like receptor family pyrin domain containing 3
NO	Nitric oxide
PI3K	Phosphoinositide 3-kinase
PT	Preferred term
ROR	Reporting odds ratio
STAT3	Signal transducer and activator of transcription 3
T2DM	Type 2 diabetes mellitus
TGF-β1	Transforming growth factor beta 1
TGR5	Takeda G protein-coupled receptor 5
TNF-α	Tumor necrosis factor alpha
UC	Ulcerative colitis
