# Supplementary material for: Prioritizing Antidiabetic Drugs for Inflammatory Bowel Disease Through Inverse Signal Detection: A FAERS Pharmacovigilance Study
Source: J Clin Med. 2026 Jun 16;15(12):4672. doi: 10.3390/jcm15124672 (PMC13301378; doi:10.3390/jcm15124672)
Supplement: Supplementary file 1 [file jcm-15-04672-s001.zip › jcm-4336342-supplementary.pdf]

Supplementary Table S1. MedDRA v24.0 preferred terms (PTs) and associated lower-level terms (LLTs) used for case retrieval

Preferred Term (PT)	**Associated Lower-Level Terms (LLTs)**
Ulcerative colitis	Acute ulcerative colitis<br>Backwash ileitis<br>Colitis ulcerative<br>Colitis ulcerative acute episode<br>Colitis ulcerative aggravated<br>Enteritis ulcerative<br>Enterocolitis ulcerative<br>Left-sided ulcerative (chronic) colitis<br>Other ulcerative colitis<br>Proctocolitis ulcerative<br>UC*****<br>UC aggravated<br>Ulcerative (chronic) enterocolitis<br>Ulcerative (chronic) ileocolitis<br>Ulcerative calcitis*****<br>Ulcerative colitis<br>Ulcerative colitis relapse<br>Ulcerative colitis, unspecified<br>Ulcerative enterocolitis<br>Universal ulcerative (chronic) colitis
Crohn's disease	Crohn's<br>Crohn's aggravated<br>Crohn's colitis<br>Crohn's disease<br>Crohn's disease (colon)<br>Crohn's disease acute episode<br>Crohn's disease aggravated<br>Crohn's disease relapse<br>Crohn's duodenitis<br>Crohn's enteritis<br>Crohn's ileitis<br>Crohn's ileocolitis<br>Crohns disease aggravated<br>Disease Crohns<br>Fistulising Crohn's disease<br>Fistulizing Crohn's disease

	Granulomatous colitis<br>Granulomatous ileitis<br>Granulomatous ileocolitis<br>Ileitis regional<br>Regional enteritis<br>Regional enteritis of large intestine<br>Regional enteritis of small intestine<br>Regional enteritis of small intestine with large intestine*****<br>Regional enteritis of unspecified site<br>Regional ileitis aggravated

Abbreviations: LLT, Lower-Level Term; MedDRA, Medical Dictionary for Regulatory Activities; PT, Preferred Term; UC, ulcerative colitis.

Note: In OpenVigil, selection of a Preferred Term (PT) automatically includes all associated Lower-Level Terms (LLTs) within the MedDRA hierarchy.

Terms marked with ***** represent noncurrent LLTs retained in MedDRA for historical data retrieval and legacy analyses.
